# P-116. Clinical Factors that affect health outcomes of pediatric patients diagnosed with Pott's Puffy Tumor: A Retrospective Cohort Study

**DOI:** 10.1093/ofid/ofaf695.344

**Published:** 2026-01-11

**Authors:** Anna E Heilers, Guliz Erdem

**Affiliations:** The Ohio State University College of Medicine, Columbus, OH; Nationwide Children's Hospital, Columbus, OH

## Abstract

**Background:**

Potts Puffy Tumor (PPT) is a rare frontal bone osteomyelitis that arises in pediatric patients. It is typically associated with recurrent sinus and ocular infections. Patients typically present with a tender central forehead bulge, rhinorrhea, fever, and orbital edema. Treatment consists of broad-spectrum antibiotics, and patients may require endoscopic sinus surgery, a craniotomy, or a combined approach with both surgical techniques to drain the abscess. As of 2001, only 21 cases had been published in scientific literature since the beginning of the antibiotic era, but we have identified 49 cases of Pott’s Puffy Tumor that were diagnosed at Nationwide Children’s Hospital alone since the beginning of 2015.

**Methods:**

Data for this project was pulled from the Nationwide Children’s Epic Platform using the Slicer Dicer Tool. Patients included in this study were found to have an encounter diagnosis consistent with the SnoMed Code for Pott’s Puffy Tumor. Inclusion criteria for this study included age less than or equal to 18. Patients were excluded if they had existing intracranial infection or trauma reported in the twelve months prior to diagnosis. This retrospective cohort study examined the relationship between clinical factors and health outcomes during the follow-up period. Data was collected by chart review in Epic. Data was analyzed in R version 4.2.1.

**Results:**

We found that out of our 49 patients, 39 (79.6%) were male and 10 (20.4%) were female. 38 (77.5%) patients underwent at least one form of surgical intervention. Additionally, we noted that there was a marked increase in the number of cases of PPT in recent years. From 2015-2019, Nationwide Children's Hospital was seeing an average of 2-3 patients with PPT per year, but from 2021-2024, Nationwide Children's Hospital was averaging 8-9 cases per year. Complications included thrombosis, infarcts, and visual disturbances. Patient requiring surgeries had prolonged hospitalization. There were no deaths.

**Conclusion:**

These findings further highlight the need for research into the clinical and surgical outcomes of Pott's Puffy Tumor. With the recent increase in PPT cases, it is essential that research continues to improve patient outcomes.

**Disclosures:**

All Authors: No reported disclosures

